# Acute Lymphoblastic Leukemia Experience: Epidemiology and Outcome of Two Different Regimens

**DOI:** 10.4084/MJHID.2013.024

**Published:** 2013-04-10

**Authors:** Salah Abbasi, Faten Maleha, Muhannad Shobaki

**Affiliations:** 1 Department of Internal Medicine and Medical Oncology, King Hussein Cancer Center, Amman, Jordan.; 2 Department of Nursing, King Hussein Cancer Center, Amman, Jordan.

## Abstract

**Objectives:**

Accurate data about adult acute lymphoblastic leukemia (ALL) are lacking. We aim to assess demographics, prognostic factors, and outcome of ALL therapy at King Hussein Cancer Center (KHCC) in Jordan, and to compare the efficacy of two protocols.

**Methods:**

We reviewed medical records of adults diagnosed and treated for ALL at KHCC from January, 2007 to December, 2011.

**Results:**

Over a 5-year period, 108 patients with ALL were treated (66 with the Hyper-CVAD regimen, and 42 with the CALGB 8811 regimen). Median age at diagnosis was 33 years, with 63% males. The most common immunophenotype was CD10-positive common ALL, and 16% have BCR-ABL translocation. Complete response (CR) rate was 88%. After a median follow-up of 32 months (range, 10–72 months), the median survival (MS) was 30 months, and CR duration (CRD) was 28 months. In the multivariate analysis, the presence of BCR-ABL translocation was the only poor prognostic factor with lower MS of 23 months (p<0.01). There was no difference in MS or CRD between the two used regimens.

**Conclusion:**

International protocols for adult ALL were successfully applied to our patients. There is no difference in efficacy between Hyper-CVAD and CALGB 8811 regimens. Future protocols for adult ALL should incorporate new targeted agents and minimal residual disease monitoring to improve outcome.

## Introduction

The estimated worldwide annual incidence of adult acute lymphoblastic leukemia (ALL) is about one in 100,000. Contrary to childhood ALL, in which modern regimens produce complete remission (CR) rates around 90%, and overall survival of more than 80% at 5 years,^1^ therapeutic progress in adult ALL has been slow, with an average survival of 20–35% in patients 18 to 60 years of age.^2^,^3^ This poor survival is in spite of the high CR rates of 75–80% in adults with ALL.

ALL requires a complex and highly diversified treatment because of its wide clinic prognostic heterogeneity. Prognosis is associated with host and disease characteristics including, age, performance status, organ function, leukemia-cell phenotype and karyotype, and the rapidity of leukemia-cell clearance and achievement of CR.^4^,^5^

There is no standard induction regimen in adult ALL, but two previously reported and widely accepted regimens have been used to treat adults with ALL at King Hussein Cancer Center (KHCC) in Jordan. The first one is the Hyper-CVAD (HCVAD) regimen, which produce CR rate of 91%, median survival (MS) of 35 months, and 5-year survival rate of 39%.^6^ The second one is the Cancer and Leukemia Group B Study 8811 protocol (CALGB regimen), which produce CR rate of 85%, and MS of 36 months.^7^

In this study, we report the demographics and characteristics of Jordanian adults with ALL, and the outcome of therapy at a single institution (KHCC) which accounts for more than 75% of cases treated in Jordan. Also, we compare the efficacy of the two different regimens (HCVAD versus CALGB).

## Patients and Methods

### Study Group

The study protocol and the procedures followed were approved by the institutional review board at KHCC, and in accordance with Helsinki declaration.

From January 2007 through December 2011, 108 adult patient (> or = 18 years of age) with newly diagnosed ALL, were treated according to the HCVAD or the CALGB protocol. Data were retrospectively collected from their charts. Patients with mature B-cell ALL (Burkitt) were excluded and patients with ALL who were treated with supportive care alone or low intensity chemotherapy were not included in the analysis. Data included, age at diagnosis, performance status, presence of organomegaly at diagnosis (splenomegaly, hepatomegaly, and/or lymph node enlargement), central nervous system (CNS) involvement at diagnosis, presence of large mediastinal mass, WBC count, platelet count, hemoglobin level, lactic dehydrogenase (LDH) level, immunophenotyping, presence of myeloid markers, karyotype, and presence of BCR-ABL translocation (Ph-positive) by fluorescence insitu hybridization analysis (FISH).

### Treatment

All treated patients at KHCC received first line therapy with either the HCVAD regimen (detailed protocol is previously described and published),^6^ or the CALGB regimen (detailed protocol is previously described and published).^7^ Patients with Ph-positive ALL below the age of 50 years, who were candidates for allogeneic stem-cell transplantation (SCT) and had a matched donor, underwent SCT as soon as possible in CR. In the period from July, 2009 to December, 2010, all patients (regardless of risk status or karyotype) who were below 40 years of age, candidate for SCT and had a matched related donor underwent SCT as soon as possible in CR. All patients with Ph-positive ALL, received imatinib mesylate during induction, consolidation, and maintenance.

### Response criteria and statistical methods

Response criteria were previously described.^3^ CR required normalization of peripheral counts with no more than 5% marrow blasts, and resolution of all extramedullary disease. Survival was calculated from the date of initiation of therapy to date of death or last follow up visit. CR duration (CRD) was calculated from the date of achievement of CR until relapse, death while in CR or last follow up visit while in CR. Data from patients undergoing SCT were not censored at the time of transplantation and were evaluated according to eventual outcome.

Differences in CR rates were analyzed using Fisher’s exact test. Survival and CRD distributions were estimated using the Kaplan-Meier method and compared using the log-rank test. P-value of 0.05 or less was considered statistically significant. All analysis was carried out using SAS version 9.1 (SAS Institute Inc., Cary, NC).

In accordance with the study objectives, the prognostic significance of age, WBC count, platelet counts, mediastinal mass, organomegaly, CNS involvement, immunophenotype, cytogenetic, and molecular analysis were assessed. The influence of these variables on CRD and survival was analyzed using a step wise Cox proportional hazards regression model.

## Results

### Patients’ characteristics

Characteristics of the 108 treated patients are summarized in Table 1. There was no difference between the two study groups. Median age was 33 years. A total of 40 patients (37%) were females. Splenomegaly was present in 27%, hepatomegaly in 12%, and lymph nodes enlargement in 38%. Median WBC count was 9.6 × 109/L, median hemoglobin level was 9.0 g/dL, and median platelets count was 67 × 109/L. CNS leukemia involvement was documented in 11 patients (10%) at presentation. The most common immunophenotype was CD10-positive common ALL in 74 patients (68%). Myeloid markers were positive in 18 patients (29% of the evaluable patients). Thirteen patients (16% of the 79 patients with assessable karyotype) had Ph-positive ALL.

### Treatment results

Ninety five of the 108 patients achieved CR (88%) with first line induction. Six patients (5%) died during remission induction, and seven patients (7%) had resistant disease. There was no significant difference in CR rates between the two study groups (90% for HCVAD and 86% for CALGB).

After a median follow up of 32 months (range, 10–72 months), the estimated MS time for the whole group was 30 months (95%CI, 29 to 32). Among patients achieving CR, the estimated CRD was 28 months (95% CI, 24 to 31).

**Figure 1:**
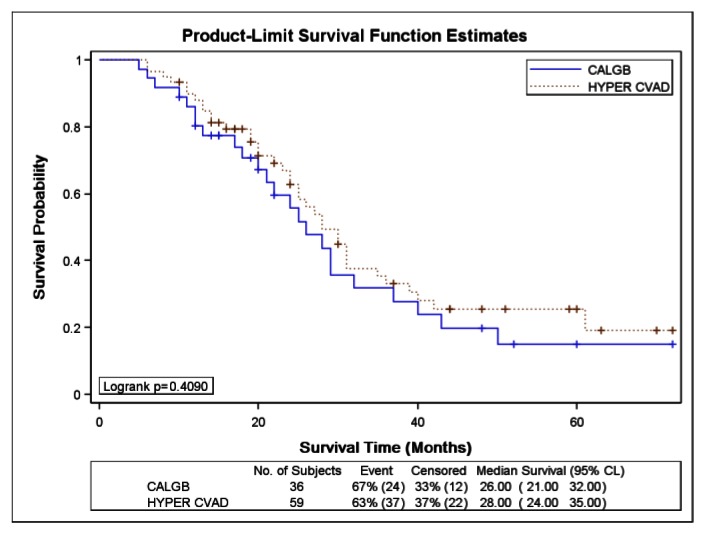
Kaplan-Meier curve for complete response duration of acute lymphoblastic leukemia patients treated with either Hyper-CVAD or CALGB 8811 regimens.

**Figure 2:**
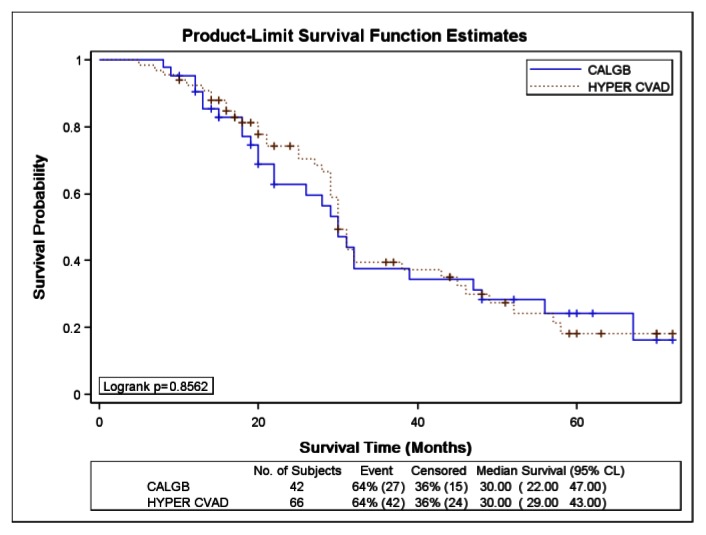
Kaplan-Meier curve for survival of acute lymphoblastic leukemia patients treated with either Hyper-CVAD or CALGB 8811 regimens

There was no significant difference in CRD or MS between the two study groups; CRD was 28 months (95%CI, 24 to 35) for HCVAD versus 26 months (95%CI, 21 to 32) for CALGB (p = 0.40) (Figure 1). MS was 30 months (95%CI, 29 to 43) for HCVAD and 30 months (95%CI, 22 to 47) for CALGB (p = 0.85) (Figure 2).

In the univariate analysis, only WBC count > 100 × 109/L at presentation and the presence of BCR-ABL translocation were associated with significantly shorter CRD and MS. In the multivariate analysis, only Ph-positive ALL was associated with significantly shorter MS; MS for Ph-positive ALL was 23 months versus 36 months for Ph-negative ALL (p < 0.01). There was no evidence of important association between outcome and sex; age; presence of anemia, or thrombocytopenia; presence of mediastinal mass, or organomegaly; CNS involvement; LDH level; immunophenotype; presence of myeloid markers; or karyotype (other than BCR-ABL translocation).

Total of 15 patients underwent Allogeneic SCT in CR1 (3 for Ph-positive ALL, and 12 others). There was no significant difference in MS or CRD between transplanted and non-transplanted patients; MS was 29 months versus 31 months, respectively (with trend in favor of non-transplanted patients, p = 0.80).

### Patients with t(9; 22)

BCR-ABL translocation was detected in 13 patients (16%) at diagnosis. All patients had CD10-positive B-cell immunophenotype. Eleven patients (85%) achieved CR1 and 2 had resistant disease. Their MS was 23 months (95%CI, 22 to 31). Only three patients were eligible for and underwent allogeneic SCT. Their ages were 29, 31, and 43 years. All transplanted patients relapsed 11, 9, and 13 months after SCT and died 16, 11, and 20 months after SCT, respectively. Among the remaining eight who achieved CR, three died in CR during consolidation, two relapsed during consolidation, and three relapsed during maintenance (including imatinib mesylate therapy).

## Discussion

The incidence, age distribution, gender, and risk categorization did not differ from those reported worldwide.^1^,^8^ Leukemia immunophenotype in our cohort of patients were similar to what is reported in the West. The incidence of BCR-ABL translocation (16%) and other genetic abnormalities were reported (Table 1). Geographic heterogeneity has been reported with regard to the frequency of t (9; 22) in ALL,^9^ which is lower in developing countries for unknown reasons. Our study shows; that the incidence of this bad translocation is not as high as published data from Western and developed countries, but not lower than other parts of the world. Previous study from Jordan showed an incidence of 7.4% in children with ALL.^10^

We report in this study the outcome of two well-established regimens in the treatment of ALL in Jordanian adults, and we compare the efficacy of these two different regimens, as no previous study compared between them before. We found that our patients using either regimen can achieve a MS of 30 months, and a CRD of 28 months. These results, although slightly lower, compare very well with published data for survival in adults with ALL from Western and developed countries. We found no significant difference between the outcome of HCVAD and CALGB regimens regarding response, MS or CRD. This is a very important finding when we compare two different regimens with different chemotherapy components, doses, schedules, and CNS prophylaxis. Although, there was a trend for better CRD with HCVAD regimen, the difference was not statistically significant. This means that other approaches are needed to improve outcome in adults with ALL, other than simply intensifying the doses or including drugs like regular asparaginase. Approaches like adding new targeted therapies (the use of Rituximab in CD20-positive ALL is an example), new chemotherapy agents like polyethylene glycol formulation of asparaginase (PEG-asparaginase), and monitoring for minimal residual disease (MRD), may result in further improvement in cure rates. We found no added benefit from SCT in CR1, although number of patients is small and not enough to make conclusions.

The slightly lower survival in our patients can have several explanations. Survival rates from low and middle-income countries have not been encouraging and range from 30–40% with high relapse rates ranging from 24% - 60%.^11^ Reasons for such poor outcomes include lack of adequate supportive care and access to care, elevated cost of health care, and compliance issues. Both the financial and compliance aspects may pose great problems in our communities.^11^,^12^ Another important factor in outcome is the host. Pharmacogenetics can impact the outcome of therapy. Genes that encode drug metabolizing enzymes, transporters, or drug targets can influence efficacy and toxicity of chemotherapy. Also, increased toxic death during induction and remission could be related to co-morbidities, nutritional status, level of hygiene, access to acute care, and adequacy of supportive care facilities. Given all these limitations in Jordan as a poor to middle-income country, we were able at KHCC to achieve acceptable and competitive results.

The outcome of patients with BCR-ABL positive ALL has improved dramatically with the use of tyrosine-kinase inhibitors (TKIs) with overall survival more than 50% at 1 to 4 years.^13^–^15^ Although most patients did not undergo SCT, our study showed a MS of 23 months in this patient population using imatinib with either HCVAD or CALGB regimen. In spite of this encouraging MS, Ph-positive patients still have worse prognosis than standard risk patients, and more work is needed to better utilize TKIs (especially new generation TKIs) and SCT in these patients to improve outcome.

Translocation (1; 19) is well known to be associated with poor prognosis. We were unable to comment on its prognostic significance among our patient population because only one case was detected.

Analysis of prognostic factors suggested that some previously well-established poor prognostic factors such as degree of leukocytosis, organomegaly, CNS involvement, LDH level, or age are less important with the two regimens used here. This is in line with observations in other tumors, in which prognostic factors are treatment dependent and in which improved therapy has eliminated or minimized the effect of pretreatment variables. In accordance with previous reports,^16^,^17^ the presence of myeloid markers was not an indicator of unfavorable prognosis, and should not be considered marker of unfavorable mixed-lineage disease.

Our study has some limitations, although it is the only study which looks at ALL demographics, prognostic variables, and outcome in adults from Jordan. First, it is a retrospective study comparing two regimens for ALL with the known selection bias in such studies; choice of regimen was according to treating physician and more young patients were treated with HCVAD. Second, it included small number of patients in either arm, which could affect the statistics. Finally, it did not look at the toxicity profile and the toxic death rates during induction and consolidation for both regimens, which is an important end point when evaluating ALL therapies. This could be an important goal to look at in our future prospective studies.

## Conclusion

International protocols may be successfully applied to our patients with good results. There is no difference between HCVAD and CALGB regimens regarding efficacy, but outcome in adults with ALL need to be further improved, using new agents and careful monitoring for MRD.

## Figures and Tables

**Table 1:** Characteristics of the study groups (108 patients).

Characteristic	HCVAD group (66 patients) No. (%)	CALGB group (42 patients) No. (%)

Median age (range)	32.5(19–73)	34(18–69)

Gender:		
Male	42 (64)	26 (62)
Female	24 (36)	16 (38)

Splenomegaly	17 (26)	12 (28)

Hepatomegaly	8 (12)	5 (12)

Lymphadenopathy	26 (40)	15 (36)

CNS disease at diagnosis	6 (9)	5 (12)

Mediastinal mass	6(9)	3 (7)

WBC count (cell/ml):		
< 30,000	48 (73)	33 (79)
30,000–100,000	12 (18)	6 (14)
>100,000	6 (9)	3 (7)

Platelet count <100,000/ml	45 (68)	30 (71)

Hemoglobin level < 10 g/dL	48 (73)	32 (76)

LDH > 600 (U/L)	28 (55)*	11 (52)**

Immunophenotype:		
CD10 negative Pre-B	12 (18)	6 (14)
CD10 positive common ALL	46 (70)	28 (67)
T-cell ALL	8 (12)	8 (19)

Myeloid markers:		
Positive	11 (30)	7 (27)
Negative	26 (70)	19 (73)
Not done/unknown	29	16

Karyotype: (%, percentage of assessable patients)		
Normal	27 (52)	17 (55)
Ph-positive (by FISH)	8 (16)	5 (17)
t(1,19)	0 (0)	1 (3)
t(4,11)	1 (2)	1 (3)
t(12,21)	7 (13)	4 (13)
Isolated +8, or −6q	3 (6)	1 (3)
Hyperdiploid	2 (4)	1 (3)
Hypodiploid	1 (2)	0 (0)
Insufficient/not done	17	12

Abbreviation: CNS, central nervous system; WBC, white blood cell; LDH, lactic dehydrogenase; Ph, Philadelphia chromosome; FISH, fluorescent in situ hybridization.

* 15 unknown,

** 21 unknown.

## References

[1] Pui CH, Robison LL, Look AT (2008). Acute lymphoblastic leukemia. Lancet.

[2] Pulte D, Gondos A, Brenner H (2009). Improvement in survival in younger patients with acute lymphoblastic leukemia from the 1980s to the early 21st century. Blood.

[3] Kantarjian HM, Walters RS, Keating MJ (1990). Results of the vincristine, doxorubicin, and dexamethasone regimen in adults with standard and high-risk acute lymphocytic leukemia. J Clin Oncol.

[4] Hoelzer D, Thiel E, Loffler H (1988). Prognostic factors in a multicenter study for treatment of acute lymphoblastic leukemia in adults. Blood.

[5] Bassan R, Hoelzer D (2011). Modern Therapy of Acute Lymphoblastic Leukemia. J Clin Oncol.

[6] Kantarjian HM, O’Brien S, Smith TL (2000). Results of Treatment with Hyper-CVAD, a Dose-Intensive Regimen, in Adult Acute Lymphocytic Leukemia. J Clin Oncol.

[7] Larson RA, Dodge RK, Burns CP (1995). A Five-Drug Remission Induction Regimen With Intensive Consolidation for Adults With Acute Lymphoblastic Leukemia: Cancer and Leukemia Group B Study 8811. Blood.

[8] Pui C-H, Evans WE (1998). Acute lymphoblastic leukemia. N Engl J Med.

[9] Johansson B, Mertens F, Mitelman F (1991). Geographic heterogeneity of neoplasia-associated chromosome aberrations. Genes Chromosomes Cancer.

[11] Halalsheh H, Abuirmeileh N, Rihani R (2011). Outcome of Childhood Acute Lymphoblastic Leukemia in Jordan. Pediatr Blood Cancer.

[13] Gao YJ, Lu FJ, Wang HS (2006). Treating childhood acute lymphoblastic leukemia in a developing country 1998–2003, the experience of a single children’s hospital in China. J Pediatr Hematol Oncol.

[14] Metzger ML, Howard SC, Fu LC (2003). Outcome of childhood acute lymphoblastic leukemia in resource-poor countries. The Lancet.

[15] Bassan R, Rossi G, Pogliani EM (2010). Chemotherapy phased imatinib pulses improve long-term outcome of adult patients with Philadelphia chromosome-positive acute lymphoblastic leukemia: Northern Italy leukemia group protocol 09/00. J Clin Oncol.

[16] Ribera JM, Oriol A, Gonzalez M (2010). Concurrent intensive chemotherapy and imatinib before and after stem cell transplantation in newly diagnosed Philadelphia chromosome-positive acute lymphoblastic leukemia: Final results of the CSTIBES02 trial. Haematologica.

[17] Schultz KR, Bowman WP, Aledo A (2009). Improved early event-free survival with imatinib in Philadelphia chromosome–positive acute lymphoblastic leukemia: A children’s oncology group study. J Clin Oncol.

[19] Preti HA, Huh YO, O’Brien SM (1995). Myeloid markers in adult acute lymphocytic leukemia: Correlations with patient and disease characteristics and with prognosis. Cancer.

[20] Putti MC, Rondelli R, Cocito M-G (1998). Expression of myeloid markers lacks prognostic impact in children treated for acute lymphoblastic leukemia: Italian experience in AIEOP-ALL 88–91 studies. Blood.

